# Hospital Abuse

**Published:** 1889-12-07

**Authors:** Hugh Woods


### Hospital Abuse.

By Hugh Woods, M.D.

The defenders of the present hospital mismanagement are on the horns of a dilemma. If they admit that the hospitals and similar institutions are treating the vast numbers that their returns would indicate, when not analysed, then there can be no doubt that they are pauperising the community to an outrageous extent. Wishing, however, to combat the overwhelming statistics that have lately been collected, they contend that the returns of the numbers treated are, to a great degree, deceptive. But while they weaken our argument on the one side, they greatly strengthen it on the other. At a meeting in North London I showed that if the various institutions of the metropolis really gave full attendance to the entire number returned as patients (*i.e.*, one-third of the whole population as out-patients, and nearly one-fortieth as in-patients), such attendance costs far too much. If, however, we admit what is perfectly true, that only a few receive all the treatment they require from these institutions, we then see that the cost per head is exorbitant in the highest degree. Either the hospitals are treating well-to-do people with funds that belong to the poor, or they are spending far more money than ought to suffice for the work really done.

